# Effect of lignin content on changes occurring in poplar cellulose ultrastructure during dilute acid pretreatment

**DOI:** 10.1186/s13068-014-0150-6

**Published:** 2014-10-14

**Authors:** Qining Sun, Marcus Foston, Xianzhi Meng, Daisuke Sawada, Sai Venkatesh Pingali, Hugh M O’Neill, Hongjia Li, Charles E Wyman, Paul Langan, Art J Ragauskas, Rajeev Kumar

**Affiliations:** School of Chemistry and Biochemistry, Renewable Bioproducts Institute, Georgia Institute of Technology, 500 10th Street, N.W. Atlanta, GA 30332-0620 USA; Department of Energy, Environmental and Chemical Engineering, Washington University, 1 Brookings Drive, Saint Louis, MO 63130 USA; Center for Structural Molecular Biology and the Biology and Soft Matter Division, Oak Ridge National Laboratory, Oak Ridge, TN 37831 USA; Center for Environmental Research and Technology (CE-CERT), Bourns College of Engineering, University of California, 1084 Columbia Avenue, Riverside, CA 92507 USA; Department of Chemical and Environmental Engineering, Bourns College of Engineering, University of California, 900 University Avenue, Riverside, CA 92521 USA; BioEnergy Science Center (BESC), Oak Ridge National Laboratory (ORNL), Oak Ridge, TN 37831 USA; Department of Chemical and Biomolecular Engineering, Department of Forestry, Wildlife, and Fisheries, Center for Renewable Carbon, University of Tennessee, Knoxville, TN 37996-2200 USA

**Keywords:** Cellulose ultrastructure, Lignin content, Dilute acid pretreatment, Delignification, Enzymatic sugar release, Biomass recalcitrance

## Abstract

**Background:**

Obtaining a better understanding of the complex mechanisms occurring during lignocellulosic deconstruction is critical to the continued growth of renewable biofuel production. A key step in bioethanol production is thermochemical pretreatment to reduce plant cell wall recalcitrance for downstream processes. Previous studies of dilute acid pretreatment (DAP) have shown significant changes in cellulose ultrastructure that occur during pretreatment, but there is still a substantial knowledge gap with respect to the influence of lignin on these cellulose ultrastructural changes. This study was designed to assess how the presence of lignin influences DAP-induced changes in cellulose ultrastructure, which might ultimately have large implications with respect to enzymatic deconstruction efforts.

**Results:**

Native, untreated hybrid poplar (*Populus trichocarpa* x *Populus deltoids*) samples and a partially delignified poplar sample (facilitated by acidic sodium chlorite pulping) were separately pretreated with dilute sulfuric acid (0.10 M) at 160°C for 15 minutes and 35 minutes, respectively . Following extensive characterization, the partially delignified biomass displayed more significant changes in cellulose ultrastructure following DAP than the native untreated biomass. With respect to the native untreated poplar, delignified poplar after DAP (in which approximately 40% lignin removal occurred) experienced: increased cellulose accessibility indicated by increased Simons’ stain (orange dye) adsorption from 21.8 to 72.5 mg/g, decreased cellulose weight-average degree of polymerization (DP_w_) from 3087 to 294 units, and increased cellulose crystallite size from 2.9 to 4.2 nm. These changes following DAP ultimately increased enzymatic sugar yield from 10 to 80%.

**Conclusions:**

Overall, the results indicate a strong influence of lignin content on cellulose ultrastructural changes occurring during DAP. With the reduction of lignin content during DAP, the enlargement of cellulose microfibril dimensions and crystallite size becomes more apparent. Further, this enlargement of cellulose microfibril dimensions is attributed to specific processes, including the co-crystallization of crystalline cellulose driven by irreversible inter-chain hydrogen bonding (similar to hornification) and/or cellulose annealing that converts amorphous cellulose to paracrystalline and crystalline cellulose. Essentially, lignin acts as a barrier to prevent cellulose crystallinity increase and cellulose fibril coalescence during DAP.

## Background

Grass and woody biomass is mainly composed of three biopolymers, namely cellulose, hemicelluloses, and lignin, which are largely located in secondary cell walls [1]. Cellulose (typically 40 to 50 wt% of the cell wall) is a linear polysaccharide that consists of repeating β-1,4-glycosidic units and that forms both crystalline and amorphous morphologies. These cellulosic morphologies are the basis for a framework of microfibrils further associated into bundles through strong intermolecular hydrogen bonds [2]. Hemicellulose (typically 15 to 25 wt.% of the cell wall), another polysaccharide, is typically a shorter and highly branched heteropolymer composed of both 5- and 6-carbon monomeric sugars [3]. Lignin (typically 10 to 30 wt.% of the cell wall) is derived from hydroxycinnamyl monomers with various degrees of methoxylation forming a racemic, cross-linked, and highly heterogeneous aromatic macromolecule. Lignin and hemicellulose is embedded between and around cellulose microfibrils, providing rigidity and structural support to the plant cell wall [4]. Lignin, considered as the essential ‘glue’ holding cellulose and hemicellulose together, is one of the most recalcitrant components of the major plant cell wall biopolymer. The plant cell wall is described as existing as a multi-component structure that is hierarchical, with order existing on multiple length-scales. This multi-component structure forms an encapsulating matrix of lignin and hemicellulose, confining cellulose and restricting the bioavailability of cellulose for biofuels generation [5]. Also, covalent cross-linking between carbohydrates and lignin, known as lignin carbohydrate complexes (LCCs), could act as additional sites confining cellulose and promoting cell wall recalcitrance [6,7].

The biochemical conversion of biomass to biofuels involves three essential steps: pretreatment to reduce the inherent plant cell wall recalcitrance, enzymatic hydrolysis to deconstruct polysaccharides into fermentable sugars, and fermentation to convert those sugars into ethanol [8]. The main challenges related to large-scale biochemical conversion involve considerable cost and inefficiency related to enzymatic deconstruction of polysaccharides embedded in the complex structure of the plant cell wall [9], which were designed over millions of years of evolution to resist enzymatic and chemical attack. As a result, effective enzymatic hydrolysis is closely related to plant cell wall physiochemical features including cell wall chemistry and composition, cellulose ultrastructure, enzymatic accessible surface area, and so forth [10-13].

In particular, the literature has consistently cited cellulose ultrastructure, mainly crystallinity index (CrI) and degree of polymerization (DP), as a relevant performance indicator of enzymatic hydrolysis [14]. However, the exact role of cellulose CrI and DP on enzymatic hydrolysis is still not clearly defined due to the fact that biomass recalcitrance is a complex, multi-variant or scale phenomenon. For example, Del Rio *et al*. in a study on the enzymatic hydrolysis of organosolv-pretreated softwood materials demonstrated single substrate characteristics such as fiber length, cellulose DP, and cellulose CrI have little effect on an enzymatic hydrolysis yield or rate [15]. However, a variety of studies show low cellulose CrI substrates have high enzymatic digestibility; one in particular by Hall *et al*. demonstrated this on microcrystalline cellulose [16]. However, a study by Foston and Ragauskas seems to indicate that despite increases in cellulose CrI, dilute acid pretreated poplar and switchgrass showed highly increased enzymatic yields [17]. Despite these differing conclusions on the effect of cellulose ultrastructure, it is quite clear that cellulose CrI and DP are altered during pretreatment and can affect biomass recalcitrance [14,18].

The goal of pretreatment is therefore to modify plant cell wall physiochemical features such that the resulting biomass is more amenable to enzymatic deconstruction. The major effect of dilute acid pretreatment (DAP) on lignocellulose is the hydrolysis of hemicellulose and redistribution of lignin which, on average, causes beneficial (to enzymatic deconstruction) changes in cell wall chemistry and composition as well as enzymatic accessible surface area. [19,20] DAP. However, it also causes changes in cellulose ultrastructure, which most likely are deleterious (to enzymatic deconstruction) including: increases in the relative cellulose I_β_ and paracrystalline content, increases in cellulose CrI, and increases in cellulose crystallite dimension [17]. The increase in relative cellulose I_β_ content during DAP is accompanied by a decrease in relative cellulose I_α_ content, which has been attributed to a thermal transformation of the cellulose I_α_ allomorph [17,21]. Cellulose I_α_ has a meta-stable triclinic one-chain crystal structure, whereas cellulose I_β_ has a monoclinic two-chain crystal structure and is the more thermodynamic-favored crystal structure generated upon annealing at high temperatures [22-25]. DAP also produces characteristic increases in the content of paracrystalline cellulose and simultaneous decreases in the relative proportion of amorphous cellulose. The above phenomena in conjunction with decreases that occur in cellulose DP during DAP (until a ‘level-off’ DP is reached) suggest that preferential degradation or removal of amorphous cellulose, and/or a hydrothermal annealing-like process, orders amorphous cellulose and expands cellulose crystallite dimensions [26]. Nuclear magnetic resonance (NMR) spectroscopy [17] and X-ray diffraction (XRD) [27,28] results also show increases in cellulose crystallite dimension with DAP, which has been attributed to an irreversible process of cellulose co-crystallization and described as similar to hornification. In both cases, changes occurring in cellulose ultrastructure upon pretreatment require elevated temperatures and seem to be related to overcoming a kinetic barrier.

Lignin can act as a physical barrier, encapsulating and confining cellulose. This impacts enzymatic digestibility negatively [29,30]. However, if hydrothermal annealing of cellulose or cellulose co-crystallization processes are the major drivers for deleterious change in cellulose ultrastructure, the presence of lignin during pretreatment could retard those changes from occurring. This suggests, in the absence of an intact hemicelluloses and lignin matrix at pretreatment (elevated hydrothermal) conditions, that: removal of the lignin barrier layers causes cellulose crystallites to have an increased propensity to co-crystallize and coalesce through irreversible hydrogen bonding; removal of the lignin barrier layers and lack of spatial confinement causes cellulose crystallites to have an increased propensity to expand through cellulose annealing and conversion of amorphous to crystalline cellulose; and/or removal of the lignin protecting layers causes the pretreatment severity experienced by the cellulose to be higher, not altering, but simply increasing the effect of pretreatment on cellulose ultrastructure. In combination with previous studies about the complete delignification resulting in the lower sugar release [30], this finding could have significant implications, for example, on how genetically modified low-lignin plants are pretreated for enzymatic hydrolysis. Also, establishing a correlation between lignin content and the changes that occur in cellulose ultrastructure during pretreatment could help elucidate the mechanisms that are responsible for cellulose CrI and crystallite size increase during DAP. In order to investigate the effect of lignin content within the presence of an intact lignin-carbohydrate complex has on changes occurring to cellulose ultrastructure during DAP, and consequently enzymatic hydrolysis, *Populus trichocarpa* x *Populus deltoides* substrates with controlled lignin contents were prepared and then pretreated.

## Results and discussion

In an effort to assess potential cell wall compositional and chemistry changes occurring during delignification and DAP, especially those associated with biomass degradation and consequently to changes in enzymatic hydrolysis, cell wall compositional analysis was conducted.

### Cell wall compositional analysis

**Figure 1:**
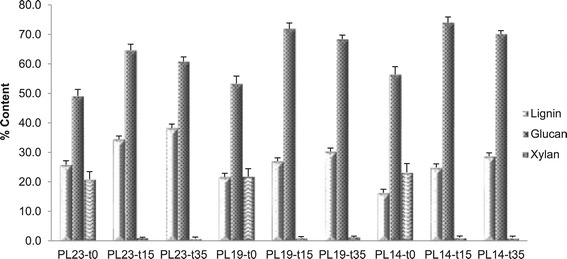
**Klason lignin, glucan, and xylan contents from dilute acid pretreated poplar with reduced lignin contents.** Sample code with definition is in Table 1.

Carbohydrate and Klason lignin (K-lignin) content for the untreated, delignified, and dilute acid pretreated poplar solids are reported in Figure 1. DAP as a cost-effective pretreatment method that significantly reduces lignocellulosic recalcitrance by removing hemicellulose, disrupting lignin-hemicellulose matrix, and redistributing lignin [17]. Delignification (holocellulose pulping) of the native poplar with starting K-lignin of about 23 wt% (Table 1 PL23-t0; t indicates DAP time in minutes) for 15 minutes resulted in a K-lignin content of about 19 wt% (PL19-t0 sample) and increased the relative glucan and xylan contents in the residual solid from 49 to 56% and 22 to 23%, respectively. Further, delignification for an additional 15 minutes dropped lignin content to about 14 wt% (to produce the PL14-t0 sample), however, there was little change in the relative glucan and xylan contents. Based solely on this data, it seems reasonable to suggest that limited delignification had little effect on the cell wall carbohydrate components.

**Table 1:** **Pretreatment methods and conditions of poplar**

**Sample code**	**Poplar sample**	**Starting % Klason lignin**	**Pretreatment conditions**
PL23-t0	Native	23.2	–
PL23-t15	Dilute acid pretreated	23.2	0.1 M H_2_SO_4_,160°C, 15 minutes
PL23-t35	Dilute acid pretreated	23.2	0.1 M H_2_SO_4_,160°C, 35 minutes
PL19-t0	Delignified	19.2	–
PL19-t15	Delignified then dilute acid pretreated	19.2	0.1 M H_2_SO_4_,160°C, 15 minutes
PL19-t35	Delignified then dilute acid pretreated	19.2	0.1 M H_2_SO_4_,160°C, 35 minutes
PL14-t0	Delignified	14.3	–
PL14-t15	Delignified then dilute acid pretreated	14.3	0.1 M H_2_SO_4_,160°C, 15 minutes
PL14-t35	Delignified then dilute acid pretreated	14.3	0.1 M H_2_SO_4_,160°C, 35 minutes

When native poplar sample (PL23-t0) was subjected to DAP for 15 minutes (to produce sample PL23-t15), there was a significant reduction of xylan from 21 to 1% (PL23-t15), with a corresponding increase of the relative glucan and Klason lignin contents. When the residence time of DAP was extended to 35 minutes, the residual solids had a slightly lower relative glucan and higher relative lignin content than the solids collected after 15-minutes pretreatment. This could be a result of hydrolytic degradation of cellulose but also, in part, result from the re-polymerization of polysaccharide degradation products forming pseudo-lignin [31]. Delignification of poplar followed by DAP resulted in a similar initial increase in relative glucan and Klason lignin contents, but a slight decrease in relative glucan content with increasing DAP residence time. Though delignification to a greater extent, followed by DAP, seems to correspond with greater magnitudes of change.

### Fourier transform infrared (FTIR) spectroscopy analysis

Relative changes in cell wall chemistry can be extracted from various absorption bands and presented in Table 2. The normalized Fourier transform infrared (FTIR) absorption spectra of lignocellulose at a band position of 1,424 cm^−1^ is primary due to the presence of cellulose, specifically the CH_2_ scissor motion of cellulose [32-34]. A decrease in the spectral band at 1,424 cm^−1^, as well as other cellulose specific spectral bands, can be used to determine possible degradation isolated to cellulose during sodium chlorite delignification followed by pretreatment. An increase in spectral intensity at 3,340 cm^−1^ represented an increase in cellulose-hydrogen bonding and indicated possible co-crystallization. Spectral intensity at 2,900 and 1,367 cm^−1^ was attributed to C-Hbond stretching and relative decreases in those bands suggested general degradation to the biomass was occurring based on the removal of methyl and methylene groups. After DAP the reduction in the band intensity from around 1,740 cm^−1^ representing carbonyl groups associated with lignin [35], typically indicated possible cleavage of association of polysaccharide with lignin and the removal of acetylated hemicellulose. The presence of acetyl groups have long be thought to inhibit enzymatic hydrolysis, and the de-acetylation that occurred during DAP suggested hemicelluloses hydrolysis that would facilitate the cellulose hydrolysis to sugar conversion [36,37]. In all substrates after DAP, spectral intensity increased at 1,595 and 1,510 cm^−1^ representing aromatic rings [34,38], indicated the increase in Klason lignin content after DAP. The reduction of spectral intensity at 1,240 cm^−1^ in all pretreated samples was tentatively attributed to the cleavage of acetyl groups. In addition, FTIR semi-quantitative analysis can examine the relative structural change in cellulose crystalline and amorphous components. The reduction of ratio I_α_/I_β_ suggested the reduction of I_α_ and/or the increase of I_β,_ and/or cellulose crystalline allomorph transformation from I_α_ to I_β_. The increase of ratio 1,100/900 cm^−1^ plus a reduction in 900 cm^−1^ suggested that the cellulose amorphous components were degraded to some extent and that amorphous cellulose could be transformed into crystalline cellulose.

**Table 2:** **Relative changes in poplar samples after dilute acid pretreatment by Fourier transform infrared spectroscopy**

**Band position**	**Assignment** **[** 32 **-** 38 **]**	**Pretreatment conditions**								
		**PL23-t0**	**PL23-t15**	**PL23-t35**	**PL19-t0**	**PL19-t15**	**PL19-t35**	**PL14-t0**	**PL14-t15**	**PL14-t35**
3340	O-H stretching, related to cellulose-hydrogen bonds	1.7	1.6	1.5	2.1	1.9	2.2	2.1	1.8	2.3
2900	C-H stretching, related to biomass methyl/methylene group	0.8	0.8	0.9	0.9	0.8	1.0	0.9	0.8	1.1
1740	Carbonyl bonds ascribed to hemicelluloses	1.3	--	--	1.6	--	--	1.5	--	--
1595	Lignin aromatic ring stretch	0.7	0.8	0.8	0.6	0.7	0.7	0.5	0.5	0.7
1510	Lignin aromatic ring stretch	0.6	0.9	1.0	0.5	0.8	0.8	0.4	0.7	0.7
1424	CH_2_ scissor motion in cellulose	1.0	1.0	1.0	1.0	1.0	1.0	1.0	1.0	1.0
1367	Aliphatic C-H stretch in CH_3_	1.1	0.9	0.8	1.3	1.1	1.1	1.4	1.2	1.1
1265	Ester absorption associated with uronic acid	--	1.1	1.2	--	1.2	1.1	--	1.3	1.2
1240	C-O absorption from acetyl group cleavage	1.6	1.3	1.2	2.2	1.1	1.0	2.0	1.2	1.1
1059	C-O stretch in secondary alcohol	5.0	3.6	3.4	--	4.0	4.3	--	5.3	4.9
1100/900	Crystalline to amorphous cellulose ration	3.0	4.4	4.5	2.9	3.7	4.5	2.7	4.1	4.4
750/710	I_α_/I_β_	0.9	0.6	0.4	0.3	0.3	0.2	0.3	0.3	0.2
900	Amorphous cellulose	1.1	0.6	0.6	1.2	0.8	0.8	1.2	0.9	0.7

Sample code with definition is in Table 1.

### Cellulose degree of polymerization

**Figure 2:**
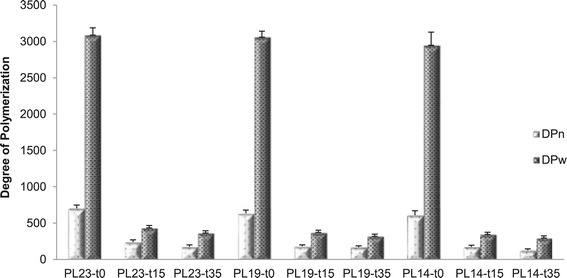
**Average number and weight degree of polymerization of cellulose.** DP_n_: number-average degree of polymerization DP_w_: weight-average degree of polymerization. Sample code with definition is in Table 1.

The cellulose average DP was determined following virtually complete lignin and hemicellulose removal and then using a published gel permeation chromatography (GPC) procedure [17]. The cellulose DP results were used to determine the ratio of terminal to interior β-glucosidic bonds that can be effectively used to analyze the relative change in cellulose chain length. Figure 2 shows the effect delignification and DAP had on poplar cellulose number-average DP (DP_n_) and weight-average DP (DP_w_). Delignification alone had a very limited effect on either DP_n_ or DP_w_, displaying at most a 5% reduction. This result is in good agreement with a previous study analyzing the effect of holocellulose pulping on cellulose molecular weight. [39] However, DAP caused dramatic reductions in cellulose DP with increased residence time. The fact that a large portion of the cellulosic component had been hydrolytically degraded during DAP could have had large implication on concurrent changes in cellulose ultrastructure, specifically the change of cellulose crystallinity. However, significant differences between cellulose DP for the sample that was subjected to DAP only and samples that was delignified with sodium chlorite followed by DAP were not detected. This is most likely a result of the cellulose reaching its leveling-off DP [17].

### Cellulose ultrastructure and crystallinity by nuclear magnetic resonance spectroscopy

In an effort to better understand the detailed ultrastructural changes occurring within cellulose during partial delignification followed by DAP, ^13^C cross-polarization (CP) magic-angle spinning (MAS) NMR spectroscopy experiments were applied to isolated cellulose to determine the relative intensity of crystalline and amorphous ultrastructural components of cellulose, following published procedures [40,41]. These results were then used to support observations made via FTIR analysis and to understand how crystalline and amorphous ultrastructural components of cellulose vary as a result of DAP and delignification followed by DAP.

**Figure 3:**
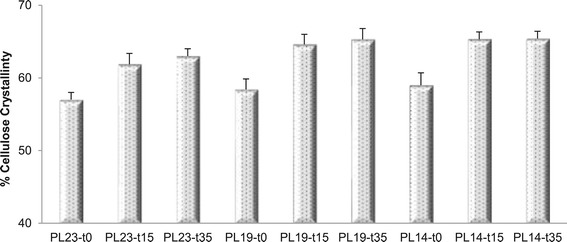
**Percent crystallinity of cellulose from dilute acid pretreated poplar with reduced lignin contents.** Sample code with definition is in Table 1.

Cellulose% crystallinity or CrI was obtained via two-peak integration of the ^13^C CP/MAS spectrum of isolated cellulose. Cellulose CrI was calculated by taking the ratio of the integral of the cellulose C_4_-crystalline carbon region (δ approximate to 85 to 92 ppm) over the integral of whole cellulose C_4_-carbon region (δ approximate to 80 to 92 ppm), and the results are shown in Figure 3. Delignification alone had little effect on cellulose CrI, in contrast, DAP generated an increase from 57 to 65%, approximately. Extended DAP residence time caused a further increase in the cellulose crystallinity, and this trend continued for samples that were subjected to delignification followed by DAP. This increase in cellulose CrI with pretreatment, in part, resulted from the preferential hydrolysis and removal of amorphous cellulose, an inference supported by the FTIR results and cellulose DP data.

**Figure 4:**
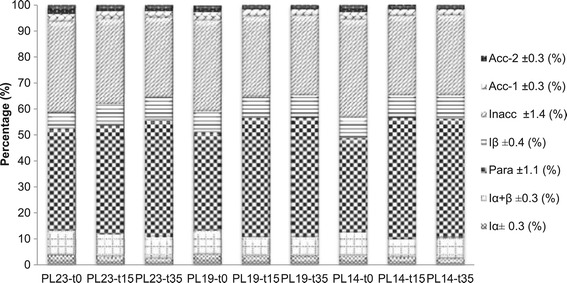
**The relative% cellulose crystalline allomorphs, paracrystalline cellulose and cellulose fibril surface.** Para: paracrystalline cellulose; Inacc: Inaccessible fibril surface; Acc-1, Acc-2: Accessible fibril surface 1 and 2. Sample code with definition is in Table 1.

The relative proportion of cellulose crystalline allomorphs, including I_α_, I_β_, and paracrystalline and amorphous cellulose at accessible and inaccessible fibril surfaces can also be extracted from the same C_4_-carbon region in the ^13^C CP/MAS spectrum of isolated cellulose using a more complex seven-peak model and a least-squared non-linear fit. The results of this analysis are shown in Figure 4. Delignification seems not to have a significant effect on cellulose ultrastructure for both crystalline and amorphous components, which is in good agreement with a previous study analyzing the effect of acidified sodium chlorite treatment on pure cellulose [30]. However, DAP caused an observed increase in relative cellulose I_β_ content, which was accompanied by a reduction in resonances representing cellulose I_α+β_ and I_α_ content, suggesting cellulose I_α_ was subject to preferential degradation and/or transformation into cellulose I_β_ during hydrothermal conditions. The latter would occur via H-bonding disruption and rearrangement under pretreatment conditions. This could be regarded as a cellulose annealing [42]. Paracrystalline cellulose is a form of cellulose that is less ordered than crystalline cellulose but more ordered than amorphous cellulose, and has been proposed to exist on the sub-surface of crystallites as a thin molecular layer [43]. Further ordering of amorphous cellulose into these paracrystalline layers could contribute to the observed increase in CrI and expansion of the crystalline lattice. All pretreated samples had a higher relative intensity for paracrystalline than the native or solely delignified samples.

Two forms of non-crystalline cellulose have been identified within the C_4_-carbon region in a ^13^C CP/MAS spectrum of isolated cellulose, amorphous cellulose at accessible and inaccessible (fibril-to-fibril contact) fibril surfaces. The relative proportion of cellulose at accessible and inaccessible fibril surfaces is also shown in Figure 4. Delignification did not alter the inaccessible fibril surfaces but slightly decreased accessible surfaces, however, DAP generated obvious reduction in inaccessible and accessible surfaces. In conjunction with GPC and crystallinity results, it could suggest hydrolysis and degradation of amorphous cellulose is kinetically favored over that of crystalline cellulose during DAP, longer residence time could cause cellulose recrystallization into crystalline I_β_, which proceeds with induced hydrogen bonding process in solvent of high polarity acidic system.

**Figure 5:**
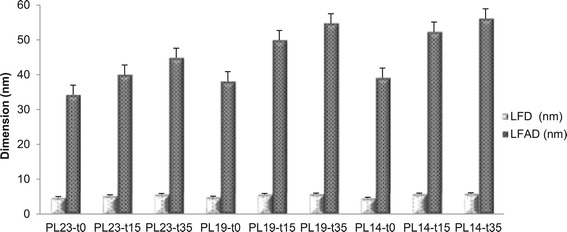
**Lateral fibril dimension (LFD) and lateral fibril aggregate dimension (LFAD) of treated poplar cellulose.** Sample code with definition is in Table 1.

In addition to the relative proportion of cellulose crystalline allomorphs and amorphous cellulose at accessible and inaccessible fibril surfaces, the C_4_-carbon region in a ^13^C CP/MAS spectrum of isolated cellulose along with a simple geometric cellulose fibril model [44,45] can estimate the average lateral fibril dimension (LFD) and lateral fibril aggregate dimension (LFAD) of cellulose. The results of this analysis are shown in Figure 5. Delignification had little effect on LFD but generated an increase in LFAD from approximately 35 to 39 nm, which could be attributed to lignin removal [46,47]. DAP seems to cause an increase in LFD and LFAD, where the extent of increase was directly correlated to the increase in pretreatment residence time. The effect of DAP on the increase of LFD and LFAD was further enhanced by greater delignification.

### Cellulose crystallite size analysis

**Figure 6:**
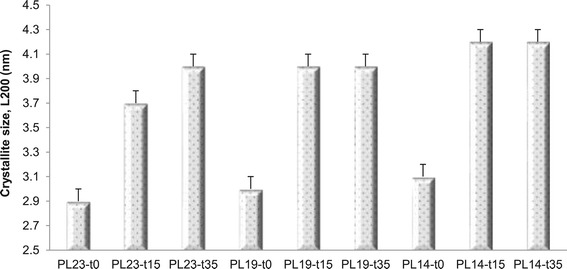
**Crystallite size (L200) for treated poplar samples.** Sample code with definition is in Table 1.

Wide-angle X-ray diffraction (WAXD), a more traditional but also complementary technique to NMR spectroscopy to extract information detailing cellulose ultrastructural features, is particularly sensitive to crystalline region due to their regular or repetitive arrangement of atoms. Two important measureable parameters are d_*hkl*_, distance between atomic planes perpendicular to (*hkl*) direction and L_*hkl*_, the distance or size of crystalline order in the (*hkl*) direction. As shown in Figure 6, delignification, DAP and delignification followed by DAP increased cellulose crystallite size to different extents. The increasing trend of cellulose crystallite size was in good qualitative agreement with the cellulose fibril dimensions extracted from NMR spectroscopy in Figure 5. DAP increased cellulose crystallite size of delignified substrates from 3.0 to 3.1 nm to 4.0 to 4.2 nm, which is interesting because scattering studies of sliced intact poplar samples indicate the cellulose fibril-fibril distance is approximately 4.0 nm [27], and any increase in the crystallite size beyond 4.0 nm implies neighboring microfibrils coalesce by expelling any interstitial biopolymer or solvent. Cellulose microfibril coalescence would be mainly reflected in a decrease of accessible cellulose surfaces, enlargement of LFADs, and the increase of cellulose% crystallinity. Moreover, the increase in the FTIR absorption bands ester linkages of covalent lactone bridges through esterification process could relate to occurred hornification [27], and the change of hydrogen-bonded hydroxyl group in poplar samples subjected to severely delignification followed by DAP also supports the cellulose crystallites growth via co-crystallize and coalesce.

### Simons’ stain

The changes of cellulose accessibility to cellulase caused by DAP and delignification followed by DAP were also evaluated to further study lignin impact on the accessible surface area of cellulose for downstream enzymatic hydrolysis. Simons’ stain testing has been used to evaluate the accessibility of a lignocellulosic substrate by applying two dyes: Direct Blue (DB) 1 and Direct Orange (DO) 15 [48]. DB 1 has a well-defined chemical formula with a molecular diameter of approximately 1 nm, whereas DO 15 is a poly-condensation product of 5-nito-*o*-toluenesulfonic acid with a molecular diameter in the range of approximately 5 to 36 nm. These two dyes absorb different wavelengths of light, have different molecular sizes, and most importantly, have different binding affinities for cellulosic surfaces. Therefore, the ratio of DO 15 and DB 1 dye (O/B) adsorbed into the biomass can be used to indicate the relative accessibility of cellulose in a lignocellulosic substrate. Arantes and Saddler [49] found that the higher the O/B ratio, the lower the protein loading required for efficient hydrolysis. However, it is also necessary to analyze the maximum amount of DO 15adsorbed especially when large amounts of the smaller DB 1 dye are adsorbed by a substrate and cause a decrease in the overall O/B ratio. In this case, there may be a significant amount of large pore and cellulose accessibility, but analysis based solely on the low O/B ratio may skew the data interpretation [19].

**Figure 7:**
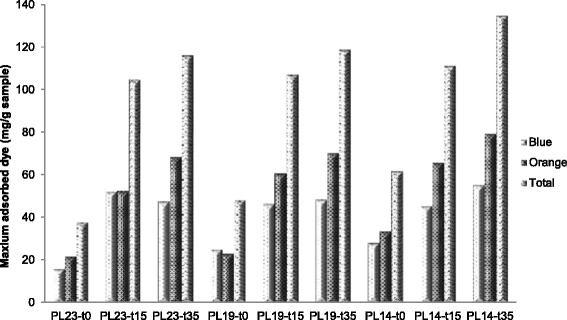
**The maximum amount of direct orange 15 dye and direct blue 1 dye adsorbed by untreated and pretreated poplar.** Sample code with definition is in Table 1.

As shown in Figure 7, the DO 15 adsorptions for samples which had not been subjected to DAP (PL23-t0, PL19-t0, and PL14-t0) were 21.8, 23.1, and 29.7 mg/g. This increase in DO 15 adsorption suggests delignification increases cellulose accessibility to some extent. For all samples under DAP for 35-minutes residence time, significant increases in the amount of DO 15 adsorbed were observed. The PL23-t0, PL19-t0, and PL14-t0 samples displayed an increase from 21.8 to 68.5 mg/g, 23.1 to 70.4 mg/g, and 28.7 to 72.5 mg/g, respectively after DAP for 35 minutes. This result indicated that DAP significantly increased cellulose accessibility such that appreciable amounts of enzymes could have access to cellulose in spite of the fact that DAP actually increases the Klason lignin content. This suggested the increased cellulose accessibility was mainly due to the hemicellulose removal [20], lignin-hemicellulose phase separation [27], and/or lignin redistribution caused by DAP.

### Enzymatic sugar release

**Figure 8:**
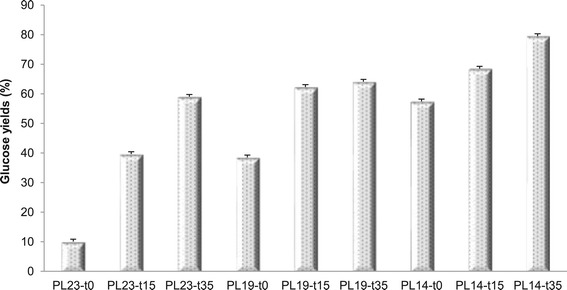
**Glucose yields as a result of downscaled enzymatic hydrolysis.** Sample code with definition is in Table 1.

Cellulose ultrastructural changes, measured as a function of pretreatment severity, were evaluated using enzymatic sugar release assays. Sugar yields were calculated through dividing the glucose contents in enzymatic hydrolysis liquid from native, delignified, and dilute acid pretreated poplar samples by the glucan contents from carbohydrate analysis on those native and treated starting materials. Figure 8 summarizes the glucose yield after enzymatic hydrolysis for the delignified substrate after DAP with respect to the unpretreated sample. DAP on undelignified substrate produced a 60% sugar yield (PL23-t35), and delignification without DAP also produced a 57.5% yield (PL14-t0). However, initial delignification followed by a second DAP step dramatically enhanced downstream enzymatic hydrolysis to facilitate sugar yields of approximately 80% for PL14-t35. In addition, P14-t0 with a lower cellulose accessibility (Figure 7) and higher sugar yield than P23-t15 suggests delignification could contribute more to the extent of enzymatic hydrolysis than DAP, as delignification enhances both enzymes macro-accessibility to cellulose and hemicelluloses, as well as enzymes effectiveness [50].

Enzymatic hydrolysis of cellulosic biomass is restricted by substrate recalcitrant factors and influenced by treatment methods. In combination with the results above, reduction in poplar cellulose DP caused by DAP could increase cellulose chain-reducing ends [51], and short chains with a weak hydrogen-bonding network could make cellulose more amenable to enzymatic deconstruction [52]. The increase of cellulose accessibility to cellulases caused by DAP is mainly due to the expansion of the pore size and volume, and the increase of a specific surface area, which thereby improve cellulases adsorption on cellulose surface [53,54]. The inevitable increase of cellulose crystallinity and crystallite size with microfibril coalescence after DAP seems to have a negative effect on enzymatic hydrolysis since crystalline regions reduce the resulting enzymatic degradation of cellulose [16]. However, some changes on cellulose crystalline allomorphic states may be beneficial to enhance the sugar yield in enzymatic hydrolysis, such as the increased proportion of paracrystalline cellulose. It is believed to be located on the surface of crystallites as thin monocellular layers which weaken the crystallites, increase cellulose dissolution and accessibility to reagents, and cause intra-lattice swelling [43]. Furthermore, studies of acidified sodium chlorite treatment on Avicel cellulose with different crystallinities have proved that those minimal changes on crystalline and amorphous cellulose by sodium chlorite had no detectable effect on cellulose digestibility [30], which suggests the major role of sodium chlorite treatment on biomass is removing lignin with intact cellulose left, and thereby enhancing the cellulose digestibility.

Lignin content and distribution had a more pronounced effect on biomass recalcitrance and enzymatic digestibility, especially for poplar [29]. However, complete lignin removal on corn stover by sodium chlorite treatment following DAP has been found to reduce cellulose conversion [30], which was proposed to be attributed to cellulose microfibril aggregation in the absence of lignin and hemicellulose. This was confirmed by our NMR and WAXD analysis that indicated lignin presence played a key role in preventing cellulose crystallites increased propensity to co-crystallize and coalesce during DAP, which therefore suggested partial delignification instead of complete lignin removal could better benefit the sugar yield. Furthermore, partial delignification with hemicelluloses removal during DAP retained cell wall spatial structure without elimination of all lignin spacer, increased specific surface area, reduced its irreversible adsorption to the enzyme [55,56], and, to limited extent, caused the cellulose fibril coalescence to provide an optimal pretreated biomass for subsequent enzymatic deconstruction.

## Conclusions

This study is another important step in providing the required data for a comprehensive analysis of biomass in an effort to optimize the integrated operations of pretreatment and enzymatic hydrolysis. In particular, key molecular features related to biomass recalcitrance, specifically cellulose ultrastructure and accessibility were, studied. In the absence of lignin spacer along with hemicellulose removal after DAP, changes occurred to cellulose ultrastructure include increases in cellulose% crystallinity, cellulose crystallite size, cellulose crystalline transformation, and cellulose accessibility accompanied by a decrease of cellulose DP. NMR and WAXD results indicated that lignin presence played a key role in preventing cellulose crystallite co-crystallization and coalescence during DAP. This indicates lignin acts as a barrier which restricts cellulose crystallinity increase and cellulose crystallite growth, and that partial delignification instead of complete lignin removal is better for enhanced sugar yield.

## Materials and methods

### Substrate and enzymes

Baseline poplar (*P. trichocarpa* x *P. deltoides*) samples were harvested between 2007 and 2008 by the National Renewable Energy Laboratory (NREL) at area 0800 at Oak Ridge National Laboratory, Tennessee, United States. Cellulase (Accellerase™ 1500, Lot number: 1681198062) and xylanase (Accellerase XY, Lot number: 4901131618) were generously provided by DuPont Industrial Biosciences (Palo Alto, California, United States).

### Poplar samples under partial delignification and dilute acid pretreatment

Poplar samples were size-reduced in a Wiley mill (Thomas Scientific, Swedesboro, NJ , United States) using a 0.250 to 0.177 mm screen. Extractives were subsequently removed by placing the biomass into an extraction thimble in a Soxhlet extraction apparatus (VWR, West Chester, PA, United States). The extraction flask was filled with 1:2 ethanol:benzene mixture (Sigma-Aldrich (St. Louis, MO, United States) (approximately 150 mL in total) and then refluxed at a boiling rate which cycled the biomass for at least 24 extractions over a 4 hour period (PL23-t0). Modified acid-chlorite delignification was used to reduce lignin content. Samples were generated by exposure of extracted baseline poplar to NaClO_2_ (0.6 g/1.00 g lignocellulosic dry solids) in acetic acid (375 mL of 0.14 M) (Sigma-Aldrich (St. Louis, MO, United States) at 70°C for about 15 minutes, followed by a further 30 minutes to generate solids with 19.2 and 14.3% Klason lignin content (sample labeled as PL19-t0 and PL14-t0, respectively, shown in Table 1). The native sample without any lignin removal and delignified samples with Klason lignin content 19.2 and 14.3% were subjected to DAP under the same conditions. Poplar samples were transferred to a 4560 mini-Parr 300 ml pressure reactor (Parr Instrument Company, Moline, IL, United States) with 0.1 M H_2_SO_4_ solutions at 5% dry solids and were then sealed. The impeller speed was set to about 100 rpm, and the vessel was heated to 160°C for 25 to 30 minutes (at approximately 6°C/min). The reactor was held at the pretreatment temperature ±2°C (approximately 0.65 to 0.69 MPa) for the specified residence time ±30 seconds (15 or 35 minutes). The reactor was then quenched in an ice bath for approximately 5 minutes. The pretreated slurry was filtered to remove the solid material and washed with an excess of deionized filtered water. The pretreated lignocellulosic samples were dried in the fume hood overnight. All yields for biomass recovered after pretreatment ranged between 75 and 85% by mass. Poplar samples under delignification and DAP at various conditions are summarized in Table 1.

### Carbohydrates and Klason lignin analysis

The extractive-free biomass samples were analyzed according to the NREL standard method for the determination of structural carbohydrates and lignin in biomass [57]. Those polymeric carbohydrates, hydrolyzed into the monomeric forms and soluble in the hydrolysis liquid, were determined by high-performance anion-exchange chromatography with pulsed amperometric detection (HPAEC-PAD) using Dionex ICS-3000 (Dionex Corp., Sunnyvale, CA, United States). Error analysis was conducted by performing carbohydrate and Klason lignin analysis three times on the untreated and pretreated samples, reported as an average with error bars are one standard deviation.

### Attenuated total reflectance Fourier transform infrared (ATR-FTIR) spectroscopy analysis of native and pretreated poplar

To investigate and quantify chemical changes in untreated and pretreated poplar samples with controlled lignin content, a PerkinElmer Spectrum 100 FTIR spectrometer with a universal attenuated total reflectance (ATR) sampling accessory (Perkin-Elmer Inc., Wellesley, MA, United States) was used. Native and pretreated poplar samples were pressed uniformly against the crystal surface via a spring-anvil, and spectra were obtained by 64 scans accumulation from 4,000 to 500 cm^−1^ at 4 cm^−1^ resolution. The ATR correction and the baseline correction were carried out orderly by PerkinElmer Spectrum software (Perkin-Elmer Inc., Norwalk, CT, United States) with the equipment.

### Sample preparation for nuclear magnetic resonance spectroscopy

Isolated cellulose was generated by first isolating holocellulose from milled biomass pulp. Holocellulose was isolated from pretreated samples by exposure to NaClO_2_ (1.30 g/1.00 g lignocellulosic dry solids) in acetic acid (375 mL of 0.14 M) at 70°C for 2 hours [17,58]. The samples were then collected by filtration and rinsed with an excess of deionized filtered water . This was repeated to ensure complete removal of the lignin component. Isolated cellulose was prepared from the holocellulose sample (1.00 g) by hydrolysis for 4 hours in HCl (100.0 mL of 2.50 M) at 100°C as reported by Foston and Ragauskas [17]. The isolated cellulose samples were then collected by filtration and rinsed with an excess of deionized filtered water, and dried in fume hood.

### Sample preparation for gel permeation chromatography

Isolated α-cellulose was generated by first isolating holocellulose from milled biomass pulp using the method described above. Isolated cellulose was prepared from the holocellulose sample (1.00 g) by extraction with a 17.5% NaOH solution (50 mL) at 25°C for 30 minutes. A total of 50 mL of deionized filtered water was then added to the NaOH solution. The extraction was continued with the 8.75% NaOH solution (100 mL) at 25°C for an additional 30 minutes. The isolated α-cellulose samples were then collected by filtration and rinsed with 50 mL of 1% acetic acid, an excess of deionized filtered water, and dried in fume hood.

### Nuclear magnetic resonance analysis of cellulose

The pre-wet NMR samples (with approximately 55% water content) were prepared with isolated cellulose packed into a 4-mm cylindrical ceramic MAS rotor (Bruker, Billerica, MA, United States). Repetitive steps of packing samples into the rotor were performed to fully compress and load the maximum amount of sample. Solid-state NMR measurements were carried out on a Bruker DSX-400 spectrometer (Bruker, Billerica, MA, United States) operating at frequencies of 100.55 MHz for carbon-13 in a Bruker double-resonance MAS probe head (Bruker, Billerica, MA, United States) at spinning speeds of 10 kHz. CP/MAS experiments utilized a 5 μs (90°) proton pulse, 1.5 ms contact pulse, 4.0 second recycle delay and 4–8 K scans. All spectra were recorded on wet samples (with approximately 55% water content), and the line-fitting analysis of spectra was performed using NUTS NMR Data Processing software (Acorn NMR, Inc., Livermore, CA, United States). Error analysis was conducted by performing three individual isolations, NMR acquisitions, and line-fit data processing [59,60].

### Gel permeation chromatography analysis of cellulose

The number-average molecular weight (M_n_) and weight-average molecular weight (M_w_) were determined by GPC after tricarbanilation of cellulose. Lignin-free cellulose (15 mg) from each sample was placed in separate test tubes equipped with micro stir bars and dried overnight under vacuum at 40°C. The test tubes were then capped with rubber septa. Anhydrous pyridine (4.00 mL) and phenyl isocyanate (0.50 mL) were added sequentially via syringe. The test tubes were placed in an oil bath at 70°C and allowed to stir for 48 hours. Methanol (1.00 mL) was added to quench any remaining phenyl isocyanate. The contents of each test tube were then added drop-wise to a 7:3 methanol:water mixture (100 mL) to promote precipitation of the cellulose derivative. The solids were collected by filtration and then washed with the methanol:water mixture (1 × 50 mL), followed by water (2 × 50 mL). The cellulose derivative was then dried overnight under vacuum at 40°C. Prior to GPC analysis the cellulose derivative was dissolved in tetrahydrofuran (1.0 mg/mL), filtered through a 0.45 μm filter, and placed in a 2 mL auto-sampler vial.

The molecular weight distributions of the cellulose tricarbanilate samples were then analyzed on an Agilent GPC SEC 1200 system equipped with four Waters Styragel columns (HR1, HR2, HR4, HR6), Agilent refractive index (RI) detector and Agilent ultraviolet detector (Waters, Inc., Milford, MA, United States) (270 nm) using tetrahydrofuran (THF) as the mobile phase (1.0 mL/min) with injection volumes of 20.0 μL. A calibration curve was constructed based on eight narrow polystyrene standards ranging in molecular weight from 1.5 × 10^3^ to 3.6 × 10^6^ g/mol. Data collection and processing were performed using Polymer Standards Service WinGPC Unity software (version 7.2.1, Polymer Standards Service USA, Inc., Warwick, RI, United States). DP_n_ and DP_w_ were obtained by dividing M_n_ and M_w_ by 519 g/mol, the molecular weight of the tricarbanilated cellulose repeat unit. All reported values for molecular weight and DP were the mean average of duplicate samples, except in the case of the untreated material which was the average of six samples for each type of cellulose.

### Wide-angle X-ray diffraction analysis of native and pretreated poplar

WAXD measurements were performed using a theta-theta goniometer PANalytical X’Pert PRO diffractometer (PANalytical in Almelo, Netherlands) with Cu *Kα* radiation (*α* =1.542 Å) operating at 45 kV and 40 mA. Beam divergence on the incident and diffracted beam paths were controlled by the programmable divergence and programmable anti-scatter slits to maintain a constant illuminated spot of 10 mm on the sample. A fixed 2° anti-scatter slit and a 10-mm wide limiting beam mask on the incident beam path; soller slits of 0.04 rad divergence on both beam paths, nickel as a beta-filter, and an X’Celerator scientific detector (PANalytical in Almelo, Netherlands) on the diffracted beam path were the other optic components. The sample, covered with a kapton film to maintain its humidity during measurements, was mounted onto the Spinner PW3064 stage (PANalytical in Almelo, Netherlands) and rotated at 7.5 rpm. Data was collected in the continuous scan mode from 5° to 90° 2 *θ*. The width of the diffraction peaks associated with specific reflecting planes (*hkl*) having a repeat spacing of *d*_*hkl*_ was used to estimate the crystallite size, *L*_*hkl*_ using the Scherrer equation.

The crystallite size (or dimension) *L*_*hkl*_ is calculated by [61,62]:

1 $$ {L}_{hkl}\kern0.5em =\kern0.5em \frac{0.9\lambda }{\beta_{hkl}\kern0.5em  cos\ \theta } $$

where *λ* is the X-ray wavelength in Å; ${\beta}_{hkl}$ is the angular full-width at half maximum intensity (FWHM) in radians of the (*hkl*) line profile; and *θ* is the scattering angle. The calculated values of cellulose microfibril crystallite size, *L*_*200*_ was obtained from 5 to 30° 2 *θ* range for all samples.

### Simons’ staining

DB 1 (Pontamine Fast Sky Blue 6BX) and DO 15 (Pontamine Fast Orange 6RN) dyes were obtained from Pylam Products Co. Inc. (Garden City, New York United States). DB 1 was used as received. Although the original staining method developed by Simons utilized both dyes as received [48], later studies suggested that only the high molecular weight fraction of the DO 15 dye was responsible for the increased affinity for cellulose, whereas the low molecular weight part had a very similar affinity for cellulose as DB 1 [63]. Therefore, an ultrafiltration of DO 15 to remove the low molecular weight part was necessary, and was done by filtering a 1% (wt/wt) solution of DO 15 through a 100 K membrane using an Amicon ultrafiltration apparatus (Amicon Inc., Beverly, Massachusetts, United States) under approximately 200 kPa nitrogen gas pressure [64]. To calculate the concentration of the DO 15 after ultrafiltration, 1.00 mL of the solution was dried in a 50°C oven for a week and the weight of the solid residue was measured. Simons’ stain was performed according to the modified procedure by Chandra *et al*. [65]. The amount of dye adsorbed by the biomass sample was determined using the difference between the concentration of the initial added dye and the concentration of the dye in the supernatant calculated by solving two Lambert-Beer law equations simultaneously.

### Down-scaled enzymatic sugar release assay

The high throughput enzymatic hydrolysis method is based on the High Throughput Pretreatment and Enzymatic hydrolysis (HTPH) design at University of California, Riverside (UCR, California, United States). In this particular study, 4.5 mg dry biomass was loaded into individual wells of a custom-built metal well plate by an automation robotics platform (Symyx Technologies, Sunnyvale, California, United States). Then, 446 μL deionized water was pipetted into all wells (8-channel pipette, 30 to 300 μL, Eppendorf, Hamburg, Germany) to achieve a solid loading of 1 wt%. Next, 39 μL of a mixture of 1 M citrate buffer (pH 4.8), sodium azide solution and enzymes was pipetted into each well (8 channel pipette, 10–100 μL, Eppendorf, Hamburg, Germany). The final hydrolysates contained 0.05 M citrate buffer (pH 4.95), and 0.2 g/L sodium azide. The resulting enzyme loading corresponded to a high 112.5 mg protein of Accellerase 1500 and 37.5 mg protein of Accellerase® XY (DuPont Industrial Biosciences, Palo Alto, CA, United States), respectively, per gram of glucan plus xylan in tested biomass. The high protein loading was applied to ensure that the substrate reactivity is not masked by the enzymes ineffectiveness as they can be strongly inhibited by the compounds (such as xylooligomers and phenols) present in the enzymatic hydrolysates. After enzyme addition, the well plate was then clamped between two stainless steel plates with a flat silicone gasket in between. The plate was then placed on its side in an incubation shaker (Multitron Infors-HT, ATR Biotech, Laurel, Maryland, United States) at 50°C for 72 hours at 150 rpm. Following enzymatic hydrolysis, the well-plate block was allowed to cool to room temperature and then opened. A sealing tape (Nunc, Rochester, New York, United States) was secured to the top of all vials and the entire well plate was centrifuged (CS-6R Centrifuge, Beckman, Fullerton, California, United States) for 20 minutes at 2650 rpm. Then, 260 μL of the clear hydrolysates solution was transferred to a polypropylene 96-well plate (Agilent, Santa Clara, California, United States) for HPLC analysis. In this part, sugar concentrations were quantified using Agilent 1200 HPLC (Agilent, Santa Clara, California, United States) equipped with an Aminex™ HPX-87H column (BioRad, Hercules, California, United States) and a refractive index detector. The column heater was set at 65°C and the detector was set at 50°C. The eluent (5 mM sulfuric acid) was used at a flow rate of 0.6 ml min^−1^.

## Abbreviations

ATR-FTIR: Attenuated total reflectance Fourier transform infrared spectroscopy
C CP/MAS NMR: ^13^C cross polarization and magic angle spinning nuclear magnetic resonance
CrI: Crystallinity index
DAP: Dilute acid pretreatment
DB: Direct blue
DO: Direct orange
DP: Degree of polymerization
DP_n_: Number-average degree of polymerization
DP_w_: Weight-average degree of polymerization
GPC: Gel permeation chromatography
HPAEC-PAD: High-performance anion-exchange chromatography with pulsed amperometric detection
HTPH: High throughput pretreatment and enzymatic hydrolysis
LCCs: Lignin carbohydrate complexes
LFD: Lateral fibril dimension
LFAD: Lateral fibril aggregate dimension
WAXD: Wide-angle X-ray diffraction
